# Fish Consumption and Advisory Awareness in the Great Lakes Basin

**DOI:** 10.1289/ehp.7980

**Published:** 2005-06-13

**Authors:** Pamela Imm, Lynda Knobeloch, Henry A. Anderson, the Great Lakes Sport Fish Consortium

**Affiliations:** Division of Public Health, Wisconsin Department of Health and Family Services, Madison, Wisconsin, USA

**Keywords:** advisory, awareness, fish, Great Lakes, sport fish

## Abstract

More than 61 million adults live in the eight U.S. states bordering the Great Lakes. Between June 2001 and June 2002, a population-based, random-digit-dial telephone survey of adults residing in Great Lakes (GL) states was conducted to assess consumption of commercial and sport-caught fish and awareness of state-issued consumption advisories for GL fish. On the basis of the weighted survey data, approximately 84% of the adults living in these states included fish in their diets. Seven percent (an estimated 4.2 million adults) consumed fish caught from the Great Lakes. The percentage of residents who had consumed sport-caught fish (from any water source) varied regionally and was highest among those who lived in Minnesota (44%) and Wisconsin (39%). Consumption of GL sport fish was highest among residents of Michigan (16%) and Ohio (12%). Among residents who had eaten GL fish, awareness of consumption advisories varied by gender and race and was lowest among women (30%) and black residents (15%). However, 70% of those who consumed GL sport-caught fish twice a month or more (an estimated 509,000 adults across all eight states) were aware of the advisories. Findings from this survey indicate that exposure to persistent contaminants found in GL fish is likely limited to a relatively small subpopulation of avid sport-fish consumers. Results also underscore the public health importance of advisories for commercial fish because an estimated 2.9 million adults living in these states consume more than 104 fish meals per year and may be at risk of exceeding the reference doses for methylmercury, polychlorinated biphenyls, and other bioaccumulative contaminants.

Consumption advisories for sport-caught fish were first issued by Great Lakes (GL) states during the 1970s. These advisories were based on findings from investigations of the methyl-mercury poisonings that had occurred in Minamata, Japan, and on fish tissue analysis. Since that time, researchers have discovered that a variety of other persistent environmental contaminants, including PCBs (polychlorinated biphenyls), DDT (dichlorodiphenyltrichloroethane), and polybrominated diphenyl ethers, had found their way into the aquatic food chain and might pose a risk to frequent consumers of large, predatory fish. Currently, health departments and/or state environmental agencies in 48 states issue consumption guidelines for local sport-caught fish—fish that is caught and not purchased.

The recent methylmercury reference dose revision from 0.5 μg/kg/day to 0.1 μg/kg/day triggered states to review their sport-fish advisories and federal agencies to assess the need for a commercial fish advisory. In 2004 the U.S. Environmental Protection Agency (EPA) and the U.S. Food and Drug Administration (FDA) jointly issued consumption advice for commercial fish that was intended to protect women of childbearing age and young children against the neurodevelopmental effects of methylmercury. It became apparent to some states that there was a need for a holistic methylmercury fish consumption advisory that combined advice forlocal sport-caught fish and commercial fish. Up until this time sport-caught fish advisories largely ignored exposures from commercial fish. Current advisories are intended to assist anglers and consumers of commercial fish in selecting fish low in chemical contaminants as part of a healthy, balanced diet.

In 2001 an estimated 1.85 million fishermen purchased licenses to fish on the Great Lakes (U.S. Department of the Interior 2002). Although this figure reflects an almost 30% decline from 2.55 million in 1991, GL sport fishing continues to be a popular recreational activity for many families. Frequent ingestion of fish from these lakes has been associated with higher body burdens of PCBs, DDT, and DDE (dichlorodiphenyldichloroethylene) (Anderson et al. 1998; Fiore et al. 1989; Humphrey 1983; Schwartz e al. 1983; Sonzogni et al. 1991; Tilden et al. 1997). These persistent contaminants accumulate in the body over time and increase the risk of a variety of health problems such as liver disease (Yu et al. 1997), reproductive (Dar et al. 1992; Weisskopf et al. 2003) and neurologic problems (Rogan and Gladen 1992), endocrine changes (Braathen et al. 2004; Persky et al. 2001), and developmental delays (Jacobson et al. 1990; Jacobson et al. 1986; Kimbrough and Krouskas 2003; Longnecker et al. 1997; Tilson and Kodavanti 1998). PCBs, DDT, and DDE have been classified as probable human carcinogens by the U.S. EPA, and sport-fish consumption has recently been associated with an increased risk of breast cancer among young, premenopausal women (McElroy et al. 2004).

Prenatal exposure to methylmercury has been associated with subtle learning delays and blood pressure changes (Grandjean et al. 1998; Sorenson et al. 1999). Methylmercury exposure during adulthood has recently been linked to higher rates of cardiovascular disease and acute myocardial infarction (Guallar et al. 2002; Salonen et al. 1995).

Until the early 1990s sport-fish consumption advisories were developed independently by each state. Development of these advisories may have been based on policy considerations as well as science. This led to confusion because neighboring states often provided different advice for the same, shared body of water. This situation was confusing for anglers and may have reduced confidence in the advisories. At the direction of the Council of Great Lakes Governors, the states that border the Great Lakes (Illinois, Indiana, Michigan, Minnesota, New York, Ohio, Pennsylvania, and Wisconsin) developed a protocol for a Uniform Great Lakes Sport Fish Consumption Advisory (Anderson et al. 1993). That 1993 advisory protocol provides information on the health benefits of fish; adverse effects of contaminants; recommended quantity, frequency, and types of fish to consume; recommended fishing locations; and preparation methods that can be used to reduce exposure to bioaccumulative contaminants such as PCBs and DDE (Anderson et al. 1993).

In 1991 the Great Lakes Sport Fish Consortium of health departments in six of the eight GL states (Michigan and Pennsylvania were not members of the original consortium) was formed and received competitive funding from the Agency for Toxic Substances and Disease Registries. In 1993–1994 the consortium conducted a random-digit-dial telephone survey of 8,306 residents of the eight GL states to evaluate their total fish and GL sport-fish consumption habits, define at-risk subpopulations, and assess the effectiveness of state-issued consumption advisories. Households were selected by a computerized random-digit-dial system, and an adult was then randomly selected among those in each household. The survey found that 50% of consumers of GL sport-caught fish were aware of the consumption advisory issued by their state of residence (Tilden et al. 1997). Awareness rates varied by gender and race and were lowest among women and minorities. Those results prompted a reevaluation of GL sport-caught fish advisory programs. Previously, information was targeted almost exclusively to anglers who were predominantly male, but in recognition that advice was not reaching women or minorities, the consortium expanded program outreach materials specificallly to include materials targeted to women of childbearing age. Although the focus of the consortium was on PCB and DDE in GL fish, each state also provided consumption advice for fish caught from inland lakes and rivers based on PCB and methylmercury fish tissue levels.

Between June 2001 and June 2002 the consortium conducted a follow-up randomized telephone survey of 4,106 adults to evaluate changes in awareness and fish consumption patterns among residents of these states. In this article we summarize findings from that survey and changes that occurred between the 1993 and 2001 surveys.

## Materials and Methods

Between June 2001 and June 2002 a population-based, random-digit-dial telephone survey of adults (≥18 years of age) residing in Indiana, Illinois, Minnesota, Michigan, New York, Ohio, Pennsylvania, and Wisconsin was conducted by the Wisconsin Survey Research Laboratory (Madison, Wisconsin). This study was designed as a follow-up to the 1993–1994 study conducted in these states and involved 4,106 adults who were randomly selected from each household. Although the same basic survey instrument was used as a follow-up to the original survey, a new random sample among adult residents of these states was drawn. The total sample size was nearly half that of the original 1993–1994 study because of funding restraints. The overall Conference of American Survey Research Organizations (CASRO) response rate was 56% (CASRO 1982).

Trained telephone interviewers used standardized questionnaires to collect information on demographic characteristics and fish consumption during the preceding 12 months. Respondents were asked about their fish consumption habits in a stepwise pattern. Those who included fish in their diets were asked about sport-caught fish ingestion, specifically, any fish not purchased that was caught by the respondent or by someone else and given to the respondent. Fish purchased at a restaurant or store did not qualify as sport caught. Sport-caught fish consumers were asked about GL sport-caught fish intake, and consumers of GL sport-caught fish were asked about advisory awareness. GL sport-caught fish included fish caught in the mouths of rivers that feed into the Great Lakes. Because of the popularity of tuna in the U.S. diet and recent concerns regarding tuna as a source of mercury exposure, this survey also included a series of questions about tuna (any type) consumption, which the original 1993–1994 survey did not include. Consumers of “commercial fish only” were defined as fish consumers who reported eating no sport-caught fish in the previous 12 months. In this article commercial fish includes any type of tuna. However, where tuna is specifically referenced, it refers only to this type of commercial fish and no other.

Statistical analysis of prevalence estimates, odds ratios, and chi-square and *t*-tests were conducted using SAS statistical software (version 9.1 for Windows; SAS Institute Inc., Cary, NC). Survey data were weighted before analysis to reflect state-specific selection probability for each household and adjusted for the number of telephone lines serving the residence using 2000 Census data. Data from each state were weighted to reflect the population age (four age groups were used) and gender distribution.

## Results

### Demographic characteristics of the sample.

Of the 4,106 Great Lakes Basin residents who participated in this random-digit-dial telephone survey, 56% were female, 86% reported their race as white, 91% were high school graduates, and 50% were ≥45 years of age (Table 1). By design, the sample was stratified such that approximately 500 residents were sampled in each of the eight GL states. Data were weighted for age, gender, and state of residence to reflect the 2000 Census demographics. Our study cohort underrepresented black residents and lower income households.

### Fish consumption.

On the basis of weighting of the survey data, > 80% of the adults living in this region had eaten some type of fish during the previous 12 months. Most of the population consumed only commercial fish (any type of fish purchased and not caught; Table 2). Nearly 70% specifically reported consumption of canned or fresh tuna, revealing the popularity of this type of commercial fish (Table 1). Fewer than one quarter (22%) had eaten any sport-caught fish, and only 7% (~ 4.2-million residents) had eaten fish that were caught from one of the Great Lakes.

Although the percentage of men and women who consume fish (any type) was nearly the same (85% vs. 83%, respectively), men were more likely to eat sport-caught fish (*p* < 0.0001) and GL sport-caught fish (*p* = 0.0064) than were women. Conversely, women were more likely to have ingested tuna than were men (*p* < 0.0001). Regardless of whether the fish was commercial or sport caught, consumption prevalence was positively correlated with household income (*p*-values < 0.0001). Consumption prevalences for “any type of fish” and tuna were correlated with education (*p* < 0.0001); however, sport-caught fish and GL sport-caught fish consumption prevalences were not correlated with educational attainment. The percentage of residents who included fish and sport-caught fish in their diets varied from state to state. Consumption of any type of fish ranged from 80% among Indiana residents to 87% among residents of Wisconsin. Sport-caught fish consumption was much more common in the Midwest than in the eastern states, ranging from 44% in Minnesota and 39% in Wisconsin to 15% in New York and 16% in Pennsylvania (Table 1).

Although the sample was too small to support extensive analysis by race or ethnicity, overall fish consumption rates were similar among black and white adults and lower among other/unknown races. This difference was statistically significant between white adults and those of other or unknown races (*p* < 0.0002). White residents were significantly more likely than black residents and residents of other/unknown races to have consumed tuna (*p* < 0.01) or sport-caught fish (*p* < 0.05). Adults reporting other/unknown races were significantly less likely to eat GL sport fish than were white or black adults (*p* < 0.05).

As shown in Table 2, most adults in these states (63%) consumed commercial fish but had not eaten any sport-caught fish during the 12-month recall period. This did not differ significantly from the 1993–1994 study (62%). Among those who ate fish, the average number of meals eaten (from all sources) ranged from 44/year among those who consumed only commercial fish to 53/year among those who had eaten sport-caught fish from the Great Lakes. Based on a *t*-test of the means of the log of the number of fish meals per year, the consumption rates reported by consumers of “commercial fish only” were significantly lower than those who included “GL sport-caught fish” in their diets (*p* = 0.044). Although the difference in means for these two fish consumer groups was less in the 1993–1994 study (46 meals/year vs. 48 meals/year), this difference also proved statistically significant when conducting a *t*-test of means of the logs (*p* =0.0007). There was, however, no statistically significant difference across time periods.

For those who ate GL sport-caught fish, the average number of GL sport-fish meals consumed per year was 13. This consumption rate was higher among men than among women (14 vs. 11, respectively), but the difference was not statistically significant.

**Figure 1:**
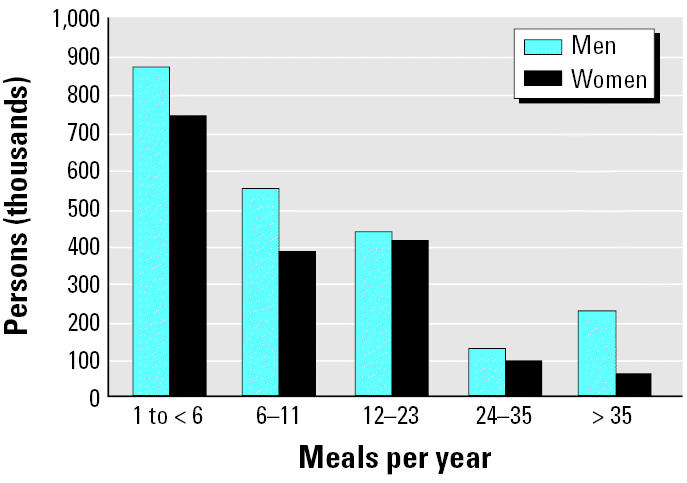
GL sport-fish meals per year among GL sport-fish consumers (weighted).

Most respondents who consumed GL sport-caught fish did so fewer than 12 times a year (range, 1–126). Depending on the type and size of the fish consumed, this rate is likely to comply with most GL fish consumption advisories. As shown in Figure 1, a small percentage of the men (10%) and women (3%) in this group ate GL fish > 35 times a year, or about 3 times a month (this gender difference was not statistically significant). Between 1993–1994 and 2001–2002, the proportion of women who ate > 35 GL sport-fish meals/year decreased significantly from 8 to 3% (*p* = 0.0418). This equates to a decrease from approximately 158,000–59,000 women who live in the Great Lakes Basin. The percentage of men in this high-consumption group did not change significantly over this time period. Overall, the estimated number of GL sport-fish consumers who ate GL sport-caught fish > 35 times per year declined from 402,000 in 1993–1994 to 286,000 in 2001–2002.

The most popular types of GL sport-caught fish were walleye number of people (*n*) = 156), perch, smelt (*n* = 152); rainbow trout, chinook, coho salmon (*n* = 139); lake trout (*n* = 121); and “other” sport-caught fish (*n* = 78). Brown trout (*n* = 40) and carp and catfish (*n* = 33) were less frequently ingested by GL sport-fish consumers.

As shown in Table 3, most adults who live in the GL states eat fish up to once a week (52 meals/year). Table 4 provides demographic descriptors and population estimates for people who consume fish more than twice a week. This subgroup comprises a high-risk population for exposure to PCBs, methylmercury, and other persistent contaminants found in large, predatory fish. As shown in Table 4, > 2.8 million residents fall into this subgroup. Most were female, college educated, and ≥ 45 years of age. People reporting a household income > $50,000/year were more likely to fall into this category than were those with lower incomes. Residents of New York were three times more likely to fall into this high-intake subgroup than were residents of any other state and approximately eight times more likely than residents of Wisconsin and Minnesota. Fifty-one percent of these high-intake individuals ate tuna at least once a week.

### Advisory awareness.

All GL states have issued consumption advisories for GL fish. Approximately half of adults who consumed fish from the Great Lakes were aware of the health advisory that had been issued by their state health department (Table 5). This awareness rate had not changed since the 1993–1994 survey. On the basis of multivariate logistic regression analysis, advisory awareness varied significantly (*p* < 0.05) by gender, black/white race, and fish consumption rate (Table 6). Whites were > 6 times more likely to be aware of their state’s advisory than blacks and men were four times more likely to be aware than women. Also, advisory awareness was positively associated with annual fish consumption rates.

Most GL fish consumers who were aware of the advisories issued by their state complied with them. Compliance rates for the types and sizes of fish that were safe to eat, preparation methods, and fishing locations ranged from 63 to 77%. The least popular recommendation was the restriction on the amount of fish that should be eaten in a given time period. Compliance with this guideline was only 52% (Table 7).

## Discussion

Although persistent, bioaccumulative contaminants in the Great Lakes Basin continue to be a public health concern, our survey results indicate that sport fishing in these lakes remains a popular activity. According to the survey results, 7% of adults living in the GL states had eaten at least one meal of GL sport fish during the previous 12 months. This percentage corresponds to an estimated population of 4.2 million adult residents in these states. Compared with national dietary estimates, residents of these states appear to consume more fish than do people living in other regions. Including residents who did not eat fish, our study revealed an average fish consumption rate of 38 meals/year. The U.S. Department of Agriculture 1994–1996 and 1998 Continuing Survey of Food Intakes by Individuals found that the average number of fish meals (grams per day converted to 6-oz prepared fish meals per year) consumed by adults ≥ 18 years of age was approximately 32/year (U.S. EPA 2002). Comparison of responses to our 1993–1994 and 2001–2002 surveys indicates that fish consumption rates and awareness prevalence have remained stable over this time period. As in 1993–1994, the 2001–2002 study suggests that most GL sport-fish consumers choose to eat fish that are low in contaminants, such as perch, smelt, and walleye.

Findings from this survey suggest that significant exposure to GL contaminants from fish is limited to a small subpopulation of avid sport-caught fish consumers. The mean number of sport-caught fish meals reported by GL sport-caught fish consumers was 13 meals/year. Only one person (a man from Michigan, 35–44 years of age) consumed more than 2 GL fish meals/week. These consumption rates are similar to those reported by respondents of the 1993–1994 survey (Tilden et al. 1997).

The overall percentage of GL sport-caught fish consumers who were aware of the advisory in their state was similar to that observed in 1993–1994. In both studies, awareness prevalence was approximately 50%. However, women and black residents reported the lowest awareness rates. In 1993–1994, less than half (38%) the women who ate GL sport-caught fish were aware that their state had issued a consumption advisory for contaminants in these fish. As a result of the 1993–1994 survey of the GL states, the health departments of these states chose as a priority to augment their ongoing activities with the development of outreach materials aimed at young women and children. This group was targeted because of methods available to reach this population and because of an emphasis on the health of this more vulnerable population because of concerns regarding the effects of fish contaminants on the developing nervous system. The first group effort produced the “Hook into Healthy Fish” campaign where the states customized a set of common outreach materials that were distributed at WIC (Women, Infants and Children) clinics and public health fairs and sent to pediatricians. These materials included coffee mugs, posters, fact cards, T-shirts, memo pads, and refrigerator magnets produced by the Wisconsin Department of Health and Femily Services, Division of Public Health and funded the the Agency for Toxic Substances and Disease Registry. Despite efforts that were intended to raise awareness among this group, from 1993 to 2002 awareness prevalence among women had slipped from 38 to 30% (although this was not a statistically significant difference). However, on a positive note, the estimated number of women who ate GL sport fish > 35 times a year declined by approximately 99,000 over this same time period. The overall effectiveness of the outreach cannot be directly evaluated because of other community and environmental action group campaigns that ran concurrently during this time period.

Awareness rates also slipped among minorities and younger age groups but rose slightly from 58 to 65% among men and continued to be highest in men ≥45 years of age (67% in 2001–2002). Although temporal changes related to awareness among these groups were not statistically significant, our findings suggest that outreach to GL fishermen and their families may not be reaching some segments of the population. This study does not allow for extensive analysis by race because of the small number of minorities who reported consumption of GL sport fish. Additional research focusing on minority populations or oversampling areas with larger minority populations is needed.

Comparisons of advisory awareness versus state of residence were not made because only a small number of respondents in each state had consumed GL sport-caught fish during the period of our study.

The need to educate the adult population about persistent toxins present in some commercial fish is evident from these survey data. Not only do most of the adult population of these GL states consume only commercial fish (63%), but also, most people in our survey who exceeded the U.S. EPA/U.S. FDA recommendation of no more than two fish meals per week consumed only commercial fish. Conversely, most of the outreach that has been conducted in these states has been targeted at licensed anglers and their families. State-issued brochures are often specific to local species and water bodies. Written advisory information, such as fishing regulation booklets and advisory brochures, has been distributed primarily to recreational fishermen and health care providers. Until recently, very little information has been available to the general public regarding contaminant levels in fish that are sold in restaurants, fish markets, and grocery stores. Although contaminant levels vary greatly among fish depending on their diets, age, and habitat, most dietary guidance has encouraged fish consumption as a healthy alternative to red meats, and advisories have limited consumption restrictions to a few highly contaminated species such as shark and swordfish. As a result, many consumers assume that all other fish is safe for unlimited consumption. Few realize that frequent, prolonged consumption of canned tuna and other predator species can lead to a high body burden of methylmercury. The U.S. FDA has published a website with methylmercury levels in commercial fish and shellfish (U.S. FDA 2004). Those with the highest levels include king mackerel, shark, swordfish, and tilefish (all close to or above 1 ppm, except king mackerel at 0.73 ppm). Other commercial fish that have average methylmercury levels above 0.5 ppm include grouper and orange roughy. Average levels in canned albacore tuna and fresh/frozen tuna are 0.35–0.38 ppm, respectively. According to recent research conducted by Hites et al. (2004), total PCBs, dioxins, toxaphene, and dieldrin levels were significantly higher in farm-raised salmon than in wild salmon. This finding is significant because more than half the salmon sold in Northern Europe, Chile, Canada, and the United States is farm raised.

Advisories that focus only on sport-caught fish miss much of the fish-consuming population. Based on our survey, > 2 million residents of the Great Lakes Basin who eat only commercial fish eat enough commercial fish to exceed safety guidelines for exposure to a variety of persistent, bioaccumulative pollutants.

## Figures and Tables

**Table 1:** Fish consumption patterns among Great Lakes Basin residents.

		% Weighted population that consumes:			
Demographic characteristic	No. of respondents (%)	Any type of fish	Tuna	Sport-caught fish	GL sport fish
Age (years)					
18–34	1,040 (25)	77	59	19	7
35–44	904 (22)	88	74	29	10
≥ 45	2,065 (50)	87	73	22	6
Gender					
Male	1,801 (44)	85	66	26	8
Female	2,305 (56)	83	72	19	6
Race					
White	3,539 (86)	85	71	23	7
Black	259 (6)	84	60	19	10
Other/unknown	302 (8)	78	59	15	4
Education					
Less than high school	386 (9)	72	54	22	7
High school graduate	1,412 (34)	80	64	23	7
Some college	995 (24)	87	72	24	8
College graduate	1,295 (32)	90	77	20	7
Household income ($)					
< 15,000	488 (12)	71	54	14	4
15,000–24,999	447 (11)	86	68	22	5
25,000–34,999	432 (11)	84	69	23	6
35,000–49,999	723 (18)	86	69	24	8
≥ 50,000	1,255 (31)	89	77	26	9
Unknown	761 (18)	81	66	18	5
State					
Illinois	508 (12)	86	68	24	5
Indiana	513 (13)	80	64	21	4
Michigan	502 (12)	82	68	25	16
Minnesota	510 (12)	84	69	44	8
New York	516 (13)	85	75	15	3
Ohio	529 (13)	83	62	21	12
Pennsylvania	508 (12)	86	71	16	3
Wisconsin	512 (12)	87	70	39	10
Total	4,106 (100%)	84%	69%	22%	7%

Percentages within groups may not total 100% because of rounding error. Percentages are based on weighted data. There were missing values for state, age, and education demographics because of partial completion of survey or refusal.

**Table 2:** Fish consumption and average number of fish meals by type of fish eaten.

Type of fish consumed	No. of respondents	% Who consume	Average no. of fish meals per year	Average no. of tuna meals per year
Commercial fish only (no sport fish)	2,442	63	44	28
Non-GL sport fish (may include commercial fish)	685	15	46	22
GL sport fish (may include commercial and/or non-GL sport fish)	299	7	53	35
None	628	16	0	0

Percentages and averages are based on weighted data. Average number of fish meals per year is calculated based on any and all types of fish, including tuna.

**Table 3:** Frequency of fish consumption by gender.

	Weighted population estimate (%)	
No. of meals per year	Men	Women
0	4,097,000 (14)	5,297,000 (17)
1 to < 12	3,452,000 (12)	4,054,000 (13)
12–24	9,074,000 (32)	8,141,000 (25)
25–52	7,762,000 (27)	9,269,000 (29)
53–104	2,960,000 (10)	3,740,000 (12)
> 104	1,342,000 (5)	1,513,000 (5)
Total	28,687,000	32,014,000

**Table 4:** Description of GL state residents who consume fish more than twice a week ( > 104 meals/year).

Demographic characteristic	*n*	Population estimate	Weighted (%)
Age (years)			
18–34	39	641,000	23
35–44	33	503,000	18
≥ 45	112	1,611,000	58
Gender			
Male	81	1,342,000	47
Female	109	1,513,000	53
Education			
Less than high school	13	219,000	8
High school graduate	38	589,000	21
Some college	45	803,000	28
College graduate	93	1,223,000	43
Household income ($)			
< 15,000	19	276,000	10
15,000–24,999	25	345,000	12
25,000–34,999	19	223,000	8
35,000–49,999	22	377,000	13
≥ 50,000	76	1,162,000	41
Unknown	29	471,000	17
State			
Illinois	24	376,000	13
Indiana	19	161,000	6
Michigan	24	324,000	11
Minnesota	14	107,000	4
New York	46	1,054,000	37
Ohio	23	352,000	12
Pennsylvania	22	342,000	12
Wisconsin	18	140,000	5
Type of consumer			
Commercial only	132	2,034,000	73
Sport fish (non-GL)	30	425,000	15
GL sport fish	25	343,000	12
Total	190	2,855,000	100

Consumption of more than two fish meals per week exceeds the U.S. EPA/U.S. FDA recommended amount. There were missing values for education and age demographics because of partial completion of survey or refusal. Type of consumer categories match those of Table 2 and are mutually exclusive.

**Table 5:** Advisory awareness among GL sport-fish consumers.

	2001–2002		1993–1994	
Demographic characteristic	*n*	% Aware	*n*	% Aware
Age (years)				
18–34	73	38	217	49
35–44	93	56	190	56
≥ 45	131	52	276	49
Race				
White	258	55	638	53
Black	24	15	38	23
Gender				
Male	153	65	355	60
Female	146	30	337	38
Education				
Less than high school	25	33	50	33
High school graduate	91	50	278	50
Some college	86	55	195	50
College graduate	97	48	165	61
Consumption				
0–5 meals/year	107	41	291	45
6–23 meals/year	135	57	248	53
≥ 24 meals/year	34	70	120	61
Total	299	49	692	51

Percentages are based on weighted data. Statistics are not provided for other races because of small sample sizes.

**Table 6:** Multivariate logistic regression model for advisory awareness among GL sport-fish consumers.

Demographic characteristic	Odds ratio (95% confidence interval)
Race	
Black	Referent
White	6.6 (2.0–21.5)
Gender	
Female	Referent
Male	4.0 (2.3–7.1)
Fish consumption	
< 6 meals/year	Referent
6–23 meals/year	2.3 (1.3–4.1)
≥ 24 meals/year	5.0 (1.7–14.6)

All odds ratios reported in table were statistically significant at *p* < 0.05 level. Regression calculated using weighted data. Statistics are not provided for other races because of small sample sizes.

**Table 7:** Self-reported compliance with advisories.

Advisory component	*n*	% Always complying
Cooking/cleaning methods	81	77
Consumption frequency	92	52
Fish species and size	65	63
Fishing locations	57	71

Percentages are based on weighted data. *n* = number of GL sport-fish consumers who reported awareness of each guideline.

## References

[b1-ehp0113-001325] Anderson HA Amrhein JF Shubat P Hesse J 1993. Protocol for a Uniform Great Lakes Sport Fish Consumption Advisory. Chicago, IL:Great Lakes Sport Fish Advisory Task Force, Council of Great Lakes Governors.

[b2-ehp0113-001325] Anderson HA, Falk C, Hanrahan L, Olson J, Burse V, Needham L (1998). Profiles of Great Lakes critical pollutants: a sentinel analysis of human blood and urine. Environ Health Perspect.

[b3-ehp0113-001325] Braathen M, Derocher AE, Wiig O, Sormo EG, Lie E, Skaare JU (2004). Relationships between PCBs and thyroid hormones and retinol in female and male polar bears. Environ Health Perspect.

[b4-ehp0113-001325] CASRO 1982. Report of the CASRO Task Force on Completion Rtes. Port Jefferson, NY:Council of American Survey Research Organizations.

[b5-ehp0113-001325] Dar E, Kanarek MS, Anderson HA, Sonzogni WC (1992). Fish consumption and reproductive outcomes in Green Bay, Wisconsin. Environ Res.

[b6-ehp0113-001325] Fiore BJ, Anderson HA, Hanrahan LP, Olson LJ, Sonzogni WC (1989). Sport fish consumption and body burden levels of chlorinated hydrocarbons: a study of Wisconsin anglers. Arch Environ Health.

[b7-ehp0113-001325] Grandjean P, Weihe P, White RF, Debes F (1998). Cognitive performance of children prenatally exposed to “safe” levels of methylmercury. Environ Res.

[b8-ehp0113-001325] Guallar E, Sanz-Gallardo MI, Van’t Veer P, Bode P, Aro A, Gomez-Aracena J (2002). Mercury, fish oils, and the risk of myocardial infarction. N Engl J Med.

[b9-ehp0113-001325] Hites RA, Foran JA, Carpenter DO, Hamilton MC, Knuth BA, Schwager SJ (2004). Global assessment of organic contaminants in farmed salmon. Science.

[b10-ehp0113-001325] Humphrey HD 1983. Population studies of PCBs in Michigan residents. In: PCBs Human and Environmental Hazards (D’Itri FM, Kamrin MA, eds). Ann Arbor, MI:Ann Arbor Science Publishers, 299–310.

[b11-ehp0113-001325] Jacobson JL, Jacobson SW, Humphrey HE (1990). The effect of intrauterine PCB exposure on cognitive functioning in young children. J Pediatrics.

[b12-ehp0113-001325] Jacobson SW, Fein GG, Jacobson JL, Schwartz PM, Dowler JK (1986). The effect of intrauterine PCB exposure on visual recognition memory. Child Dev.

[b13-ehp0113-001325] Kimbrough RD, Krouskas CA (2003). Human exposure to polychlorinated biphenyls and health effects: a critical synopsis. Toxicol Rev.

[b14-ehp0113-001325] Longnecker MP, Rogan WJ, Lucier G (1997). The human health effects of DDT and PCBs and an overview of organochlorines in public health. Annu Rev Pubic Health.

[b15-ehp0113-001325] McElroy JA, Kanarek MS, Trentham-Dietz A, Robert SA, Hampton JM, Newcomb PA (2004). Potential exposure to PCBs, DDT, and PBDEs from sport-caught fish consumption in relation to breast cancer risk in Wisconsin. Environ Health Perspect.

[b16-ehp0113-001325] Persky V, Turyk M, Anderson HA, Hanrahan LP, Falk C, Steenport DN (2001). The effects of PCB exposure and fish consumption on endogenous hormones. Environ Health Perspect.

[b17-ehp0113-001325] Rogan WJ, Gladen BC (1992). Neurotoxicology of PCBs and related compounds. Neurotoxicology.

[b18-ehp0113-001325] Salonen JT, Seppanen K, Nyyssonen K, Korpela H, Kauhanen J, Kantola M (1995). Intake of mercury from fish, lipid peroxidation, and the risk of myocardial infarction and coronary, cardiovascular, and any death in eastern Finnish men. Circulation.

[b19-ehp0113-001325] Schwartz PM, Jacobson SW, Fein G, Jacobson JL, Price HA (1983). Lake Michigan fish consumption as a source of poly-chlorinated biphenyls in human cord serum, maternal serum, and milk. Am J Public Health.

[b20-ehp0113-001325] Sonzogni W, Maack L, Gibson T, Degenhardt D, Anderson H, Fiore B (1991). Polychlorinated biphenyl congeners in blood of Wisconsin sport fish consumers. Arch Environ Contam Toxicol.

[b21-ehp0113-001325] Sorenson N, Murata K, Budtz-Jorgensen E, Weihe P, Grandjean P (1999). Prenatal methylmercury exposure as a cardiovascular risk factor at seven years of age. Epidemiology.

[b22-ehp0113-001325] Tilden J, Hanrahan L, Anderson H, Palit C, Olson J, Mac Kenzie W (1997). Health advisories for consumers of Great Lakes sport fish: is the message being received?. Environ Health Perspect.

[b23-ehp0113-001325] Tilson HA, Kodavanti PR (1998). The neurotoxicity of polychlorinated biphenyls. Neurotoxicology.

[b24-ehp0113-001325] U.S. Department of the Interior 2002. 2001 National Survey of Fishing, Hunting, and Wildlife-Associated Recreation. Available: http://www.census.gov/prod/2002pubs/FHW01.pdf [accessed 19 May 2005].

[b25-ehp0113-001325] U.S. EPA 2002. Estimated per Capita Fish Consumption in the United States. Washington, DC:U.S. Environmental Protection Agency. Available: http://www.epa.gov/waterscience/fish/consumption_report.pdf [accessed 19 May 2005].

[b26-ehp0113-001325] U.S. FDA 2004. Mercury Levels in Commercial Fish and Shellfish. Rockville, MD:U.S. Food and Drug Administration. Available: http://www.cfsan.fda.gov/~frf/sea-mehg.html [accessed 19 May 2005].

[b27-ehp0113-001325] Weisskopf MG Anderson HA Hanrahan LP2003. Decreased sex ratio following maternal exposure to polychlorinated biphenyls from contaminated Great Lakes sport-caught fish a retrospective cohort study. Environ Health 10.1186/1476-069X. 10.1186/1476-069X-2-2 PMC153540 12694628

[b28-ehp0113-001325] Yu ML, Guo YL, Hsu CC, Rogan WJ (1997). Increased mortality from chronic liver disease and cirrhosis 13 years after the Taiwan “Yucheng” (“oil disease”) incident. Am J Ind Med.

